# Stability of enveloped and nonenveloped viruses in hydrolyzed gelatin liquid formulation

**DOI:** 10.1186/s12985-022-01819-w

**Published:** 2022-05-27

**Authors:** Francois Marie Ngako Kadji, Kazuki Kotani, Hiroshi Tsukamoto, Yosuke Hiraoka, Katsuro Hagiwara

**Affiliations:** 1 Biomedical Department, R&D Center, Nitta Gelatin Inc., 2-22, Futamata, Yao City, Osaka 581-0024 Japan; 2 School of Veterinary Medicine, Rakuno Gakuen University, Ebetsu City, Hokkaido 069-8501 Japan

**Keywords:** Virus, Temperature, Stability, Hydrolyzed gelatin, Formulation

## Abstract

**Background:**

The thermal stability of viruses in gelatin liquid formulations for medical research and application is poorly understood and this study aimed to examine the thermal stability of 4 enveloped and nonenveloped DNA and RNA viruses in hydrolyzed gelatin liquid formulations.

**Methods:**

Bovine herpesvirus (BHV) was used as a model virus to examine the molecular weight (MW), concentration and gelatin type and to optimize virus stability in liquid formulations at 25 °C and 4 °C. Using the model virus liquid formulation, the stability of multiple enveloped and nonenveloped RNA and DNA viruses, including parainfluenza virus, reovirus (RV), BHV, and adenovirus (AdV), was monitored over up to a 30-week storage period.

**Results:**

The BHV model virus was considered stable after 3 weeks in hydrolyzed gelatin (MW: 4000) with a 0.8 LRV (log10 reduction value) at 25 °C or a 0.2 LRV at 4 °C, compared to the stabilities observed in higher MW gelatin (60,000 and 160,000) with an LRV above 1. Based on the gelatin type, BHV in alkaline-treated hydrolyzed gelatin samples were unexpectantly more stable than in acid-treated hydrolyzed gelatin sample. All four viruses exhibited stability at 4 °C for at least 8 weeks, BHV or AdV remained stable for over 30 weeks of storage, and at 25 °C, AdV and RV remained stable for 8 weeks.

**Conclusion:**

The results demonstrated that 5% of 4000 MW hydrolyzed gelatin formulation can act as a relevant stabilizer for the thermal stability of viruses in medical research and application.

**Supplementary Information:**

The online version contains supplementary material available at 10.1186/s12985-022-01819-w.

## Background

Many viruses used for medical research or therapeutic purposes exhibit a lack of thermostability in ambient environments. Viruses can be placed in two main categories based on the presence or absence of a lipid envelope around their capsid, which classifies them as an enveloped or nonenveloped virus, respectively. The latter is more thermostable than the former group [1, 2]. The lipid envelope plays a crucial role in virus lability and typically requires a temperature lower than 4 °C for short- or long-term storage. Nevertheless, the stability of these enveloped and nonenveloped model viruses in storage formulations is poorly understood, and virus research and human infectious disease research often employ animal-derived virus models in the virus families [3].

There are two main strategies currently used to improve virus stability: cold chain storage and freeze-drying. However, these strategies create some issues, including the challenges of cost and extensive infrastructure and maintenance for cold chain transport, especially for deep freezing [4, 5]. The cost and potential loss of virus potency in the freeze-drying process or during reconstitution are due to protein destabilization, alteration of lipid layers (enveloped viruses), or occurrence of stress related to changes in the internal and external virus environment [6]. However, to improve these strategies, many excipients are used in the formulation to increase the thermal stability of viral particles, including sucrose, dextran, albumin, and gelatin. Gelatin is a bulking agent and acceptable material for medical use and can enhance virus stability at ambient temperatures. Gelatin is preferably used as a stabilizer due to its high biocompatibility, biodegradability, low immunogenicity, and low material cost [7]. Many investigators have used gelatin for decades as a stabilizer in vaccine development. For instance, it is an excipient in some vaccines approved by the FDA, including live attenuated influenza, measles mumps rubella, shingles zoster, varicella, and yellow fever vaccines under the brand names Flumist®, MMR II®, Zostavax®, Varivax®, and YF-Vax®, respectively. The contribution of gelatin to virus stability alleviates the cost and burden of transport and storage under the cold chain process, improving virus stability at ambient temperatures, including 4 °C and 25 °C.

Very few studies have reported the thermal stability of viruses in gelatin formulations. Some studies have investigated the temperature-induced aggregation of measles particles in several stabilizing excipients, including gelatin [8], or the stability in sorbitol-gelatin formulations for measles virus [9]. Another report examined the thermal stability of varicella-zoster virus in hydrolyzed gelatin [10]. However, the characteristics of the gelatin product used for thermostability evaluation in those studies are poorly reported or unknown; there is a paucity of data on the thermal stability of viruses, including for enveloped and nonenveloped viruses in gelatin liquid formulations.

The mechanism of gelatin-mediated stabilization of viral particles in liquid inoculant formulations remains broadly unknown. Gelatin is an additive that is thought to be a good stabilizer of viruses in viral transport medium [11]. Gelatin may also play a role in resistance to temperature-induced changes in viral density, which is often concluded from accelerated studies [8]. Moreover, the heterogeneous nature of gelatin makes explaining its mechanistic contribution to viral stability challenging. Many reports have speculated that gelatin might provide noncovalent binding via electrostatic interactions for virus stability [11]. In the case of cationized gelatin (introducing amine residues onto the carboxyl groups), a complex is formed with a negatively charged viral capsid or nucleic acid through electrostatic interactions [12], which appears to prevent the degradation of surface proteins relevant to virus stability during storage or for the sustained release of an encapsulated drug in the body. It is also known that gelatin viscosity not only suppresses the phase transformation but also decreases osmotic pressure in protein solutions [13], and gelatin at an optimum concentration prevents virus aggregation, thereby improving stability through mutual incompatibility and special exclusion interactions with virus particles [14]. The properties of gelatin used in previous studies on the stability of viruses are poorly known. Therefore, this study aimed to examine the thermal stability of 4 enveloped and nonenveloped DNA and RNA viruses in liquid formulations with known gelatin parameters, including concentrations, molecular weights (MWs), and gelatin types, including type A (acid-treated gelatin) and type B (alkaline-treated gelatin), to gain a greater understanding of their effects on virus stability profiles.

## Materials and methods

### Bulk virus preparation and storage

Bovine herpesvirus 1 (BHV-1) as Herpesviridae, PI-3 (bovine parainfluenza type 3) as Paramyxoviridae, Reovirus 3 (RV-3; ATCC VR-824) as Reoviridae, and human adenovirus 5 (AdV-5; ATCC VR-1516) as Adenoviridae were used as test viruses to examine virus thermal stability in gelatin liquid formulations. The BHV-1, PI-3, RV-3, and AdV-5 inoculums were prepared, and infected monolayers of MDBK cells (BHV-1 and PI-3), HEK-293 cells (AdV-5), and LLC-MK2 cells (RV-3) were cultured with Dulbecco's modified Eagle’s medium (DMEM; D6026 Merck) containing 5% fetal calf serum following incubation at 37 °C for 6–7 days post-infection. Each virus-infected culture was harvested and centrifuged at 2500 rpm for 15 min at 4 °C, and the supernatant was collected, transferred into SW41Ti Beckman UC tubes, and ultracentrifuged for 2 h at 28 K rpm to produce pellets of high titer of virus stocks. Pellets were dissolved in gelatin liquid formulations. Aliquots of 250 µL/tube were made and pretested to determine the initial virus titer, and the rest were stored at − 80 °C as references and at ambient temperatures, including 4 °C and 25 °C, for virus thermal stability evaluations.

### Gelatin samples

The gelatin samples used for formulation were of either porcine or bovine origin. They included pigskin and bovine bone gelatin resulting from acid or alkaline extraction methods. All bovine materials used for gelatin production are not from countries at risk of BSE or specified risk materials, and a process for prion inactivation is included in the gelatin manufacturing process. Specifically, the samples were beMatrix™ Gelatin series, from Nitta Gelatin Inc. (Osaka, Japan). Additionally, recombinant human serum albumin (rHSA) of molecular weight 66,000 from Albumedix (Nottingham, UK) was also used as a reference control for the viral stability test. Various MWs of hydrolyzed gelatin (HG) were also obtained from one of the original products (beMatrix™ Gelatin LS-H) through heat hydrolysis at different temperatures and pH values, and the samples were all finally adjusted to pH 7 prior to use in the formulations. The resulting MW of the samples was measured by gel permeation chromatography following the manufacturer’s protocol (Tosoh Bioscience). Briefly, a 0.2% gelatin solution adjusted with distilled water was passed through a 0.2 µm filter and vortexed. The samples were placed in the autosampler for analysis under the following conditions: a 1 mL/min flow rate, column temperature at 40 °C, 10 µL injection volume, and a 214 nm UV wavelength. The isoelectric point determination for each sample was performed using the zeta potential method with a ZetaSizer Nano ZS (Malvern Panalytical, UK). Briefly, samples were dissolved in buffer made of a 0.1 M Tris–HCl (pH 7.7–9.0) solution and acetate solution (pH 4.0–5.50) to make 0.25% (w/v). The zeta potential was measured, and the pH variation depending on the zeta potential was plotted and extrapolated to derive the isoelectric point at zero [15].

### Cytotoxicity test

To select cytotoxicity-free samples for the gelatin formulation, a total of three buffers (6 mM sodium phosphate + 150 mM NaCl + 10% glycerol [pH 7.2]; 20 mM Tris + 25 mM NaCl + 2.5% glycerol [pH 8.0]; and 6 mM sodium phosphate + 150 mM NaCl + 15% sucrose [pH 7.4]) and gelatin samples of various MWs were evaluated. Briefly, each sample was serially diluted twofold in DMEM (Dulbecco’s modified Eagle’s medium) and dispensed at 50 µL/well (triplicate) into a 96-well plate, followed by the addition of 10,000 Vero cells per well and incubation at 37 °C for 72 h. A volume of 20 µL of MTS (3-(4,5-dimethylthiazol-2-yl)-5-(3-carboxymethoxyphenyl)-2-(4-sulfophenyl)-2H-tetrazolium) from Promega (Cat #G3580) was added and incubated at 37 °C for 3 h, followed by absorbance measurement at 490 nm on a plate reader (Ultramark Microplate Reader, Bio-Rad). The survival rate was determined using the formula [Survival rate = (Sample O.D.- Blank)/(Mock OD- Blank) × 100].

### Virus titration

Virus titration was carried out before and after storage at − 80 °C, 4 °C and 25 °C for a certain number of weeks. Briefly, triplicates of aliquots for each virus sample before or after a storage period were serially diluted tenfold in cell medium; and inoculated on relevant monolayers, including MDCK cells for BHV-1 and PI-3, HEK-293 cells for AdV-5, and LLC-MK2 cells for RV-3, in 96-well plates; and incubated at 37 °C. The cytopathic effect was recorded 6 days post-infection. The virus titers were determined as the 50% tissue culture infectious dose (TCID_50_/mL) following the Karber method [16]. Viral titers were measured for the three storage solutions, and the log10 reduction value (LRV) was calculated as the difference between the log10 of preincubation virus titers and the log10 of post incubation virus titers. An LRV above 1 or over 90% reduction in titer was considered a loss of virus infectivity [17–20].

### Statistical analysis

Data analysis, including group comparisons and graphical representations, was conducted using Prism software 8.0 (GraphPad, San Diego, CA, USA), and a *P value* < 0.05 was considered statistically significant. (Each experiment was carried out in triplicate, and the bar graphs show the means and standard deviations of LRV).

## Results

GTS buffer (20 mM Tris–HCl, 2.5% glycerol, 25 mM NaCl, pH 8.0) and gelatin samples of various MWs with final concentrations ranging between 0.4% and 25% demonstrated no cytotoxicity (data not shown). In conducting the virus storage stability test, BHV-1, which had the highest decrease in stability under 5% FCS DMEM conditions at 4 °C for 4 weeks, was used as a model virus for the development of gelatin liquid formulations.

**Fig. 1:**
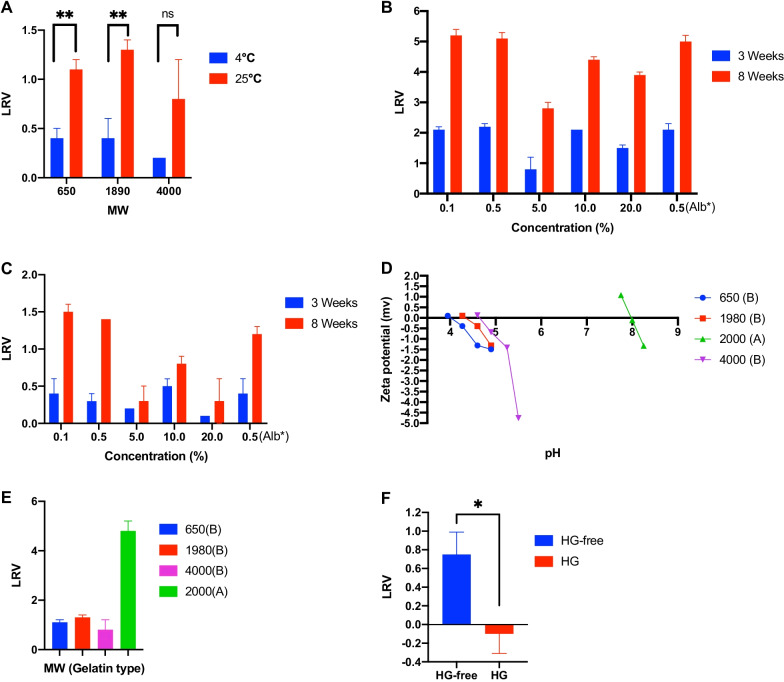
**A** Stability over 3 weeks of storage of BHV-1 in the hydrolyzed gelatin liquid formulation at 4 °C and 25 °C. LRV: Log reduction value, **P value < 0.01 (Welch’s *t* test), ns: non statistically significant. **B** Stability of BHV in the hydrolyzed gelatin liquid formulation with varying concentrations in 3- and 8-week storage at 4 °C. LRV: Log reduction value. **C** Stability of BHV in hydrolyzed gelatin liquid formulation with varying concentrations in 3- and 8-week storage at 25 °C. LRV: Log reduction value. **D** Isoelectric points of varying acid-treated and alkaline-treated hydrolyzed gelatin used for virus stability formulations. Zeta potential and pH of each molecular weight samples were showed in the figure. **E** Stability of BHV in acid-treated and alkaline-treated hydrolyzed gelatin after 3 weeks of storage at 25 °C. The numbers indicate the molecular weight of samples, and A and B, acid-treated and alkaline-treated hydrolyzed gelatin respectively. LRV: Log reduction value. **F** Effect of three freeze–thaw cycles on BHV stability in hydrolyzed gelatin. LRV: Log reduction value, *P value < 0.05 (Welch’s *t* test), HG: Hydrolyzed gelatin

In total, 5 samples with different gelatin molecular weights in the formulation in GTS buffer with BHV-1 were monitored primarily for 3 weeks at 25 °C and 4 °C. BHV-1 remained stable after 3 weeks of storage in 4000 MW hydrolyzed gelatin liquid formulation at 25 °C, in contrast to the loss of stability observed in gelatin liquid formulations with 60,000 and 160,000 MWs. (Table 1). Additionally, BHV-1 remained stable in all three HG liquid formulations at 4 °C for 8 weeks of storage. Moreover, the HG formulation with 4000 MW appeared as a better stabilizer of BHV at both 4 °C and 25 °C, showing no statistically significant difference (Fig. 1A).

**Table 1:** Three-week storage stability of BHV in the gelatin formulation with varying molecular weights at 25 °C

Gelatin MW	650	1980	4000	60,000	160,000
LRV	1.1	1.3	0.8	2.0	2.9
SD	0.1	0.1	0.4	0.3	0.2

*MW* molecular weight, *LRV* log reduction value, *SD* standard deviation

*LRV* log reduction value, *SD* Standard deviation

Hydrolyzed gelatin with 4000 MW, which demonstrated a better virus-stabilizing formulation at 4 °C and 25 °C compared to that of other HG samples, was singled out to make concentration-varying formulations of 0.1%, 0.5%, 5%, 10%, and 20% (w/w) % to find the optimal concentration for virus storage stability. In addition, 0.5% rHSA in GTS buffer was also used as a control in the same evaluation. Only the concentration of 5% showed good stability after 3 weeks of storage at 25 °C, while loss of rHSA stability occurred (Fig. 1B). Meanwhile, at 4 °C, all HG concentrations including 0.1%, 0.5%, 5%, 10%, 20% and rHSA yielded good stability after 3 weeks of storage (Fig. 1C).

Additionally, the isoelectric points of different acid-treated or alkaline-treated HG were determined to be 4.0, 4.3, 4.6 and 8.0 for HG 650 MW, 1980 MW, 4000 MW and 2000 MW respectively (Fig. 1D). The analysis of BHV-1 stability according to gelatin type was performed, and the LRV of BHV-1 in acid-treated HG was approximately 4 times greater than the LRV of BHV-1 in alkaline-treated HG at 25 °C (Fig. 1E).

To evaluate the effect of freezing and thawing samples multiple times on the stability of BHV-1, freeze–thaw stability testing was carried out, and the BHV-1 infectivity was preserved after three freeze–thaw cycles in liquid formulation with or without HG. However, BHV-1 was significantly more stable in the formulation with HG than in the formulation without HG (Fig. 1F).

**Fig. 2:**
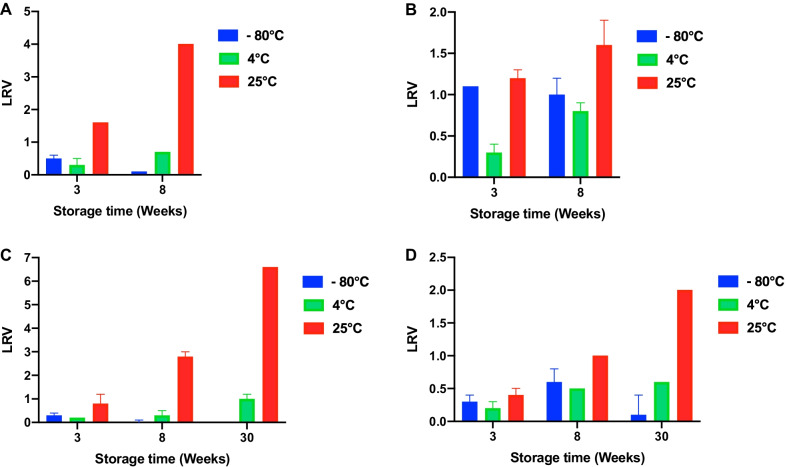
**A** Stability of PIV in the hydrolyzed gelatin liquid formulation over 8 weeks of storage at − 80 °C, 4 °C and 25 °C with a stock virus titer of 2 × 10^5.5^ TCID50/mL. LRV: Log reduction value. **B** Stability of RV in the hydrolyzed gelatin liquid formulation over 8 weeks of storage at − 80 °C, 4 °C and 25 °C with a stock virus titer of 2 × 10^5.2^ TCID50/mL. LRV: Log reduction value. **C** Stability of BHV in the hydrolyzed gelatin liquid formulation over 30 weeks of storage at − 80 °C, 4 °C and 25 °C with a stock virus titer of 2 × 10^8.1^ TCID50/mL. LRV: Log reduction value. **D** Stability of AdV in the hydrolyzed gelatin liquid formulation over 30 weeks of storage at − 80 °C, 4 °C and 25 °C with a stock virus titer of 2 × 10^8.0^ TCID50/mL. LRV: Log reduction value

A long-term 30-week storage stability study of enveloped and nonenveloped, BHV-1, and AdV-5 in hydrolyzed gelatin 4000 MW-based liquid formulation was carried out, and the results showed that the LRV was ≤ 1 at 4 °C and PI-3 and RV-3 also remained stable after 8 weeks storage (Fig. 2A-D).

## Discussion

The experiments discussed above revealed the stability of enveloped and nonenveloped viruses in gelatin liquid formulations. The gelatin liquid formulations with high MWs of 60,000 and 160,000 showed a drastic loss of infectivity, which was over a 2 LRV, compared to that of HG at 25 °C. The same experiment was not carried out at 4 °C due to the high gelling factor of high-MW gelatin. A higher molecular weight had a negative effect on virus stability, which may likely be caused by the high viscosity factor disfavoring virus particle stability in the formulation. Another possibility is the greater loss of virus infectivity during the thawing of the gelled samples at 37 °C, which required a longer time to melt due to the higher gel strength than that of HG in which there was no gelling strength and no thawing process was required after storage at 4 °C or 25 °C prior to the CPE assay. Among the HG samples, the 4000 MW sample appeared to enhance virus thermal stability at 25 °C or 4 °C compared to HG samples of lower MW, including 650 and 1980, and the concentration of 5% was found to be the optimum concentration to maintain virus stability. Hydrolyzed gelatin acts as an antioxidant and an electron donor to produce stable products prone to react with free radicals [21]. Hydrolyzed gelatin is commonly present in some vaccines at different concentrations as a stabilizer [22], and the optimal concentration may depend on the buffer or other ingredients in the formulation.

All studied viruses exhibited stability at 4 °C for at least 8 weeks, while BHV-1 and AdV remained stable for over 30 weeks of storage. In this report, BHV-1 also remained stable for 3 weeks at 25 °C showing an improved result over previous formulations. In another report, HSV stability lasted over a period of 39 weeks at 4 °C in either a 0.5% partially HG or an rHSA liquid formulation [23]. The difference observed could be explained by some factors, including the heterogeneous nature of HG, the difference in buffer compositions used in each study, the virus species used for the stability test, or most likely, the initial virus stock. BHV and HSV both belong to the same Herpesviridae subfamily; HSV belongs to the Simplexvirus genus, and BHV belongs to the Varicellovirus genus [24]. However, progeny BHV-1 appear to comprise an abundance of capsidless, noninfectious light particles, in contrast to HSV, and both present variations in tegument content [25]. BHV-1 in GTS buffer without gelatin was stable at − 80 °C (0.2 log10 reduction) following 8 weeks of storage, but not at ambient temperatures such as 4 °C or 25 °C (Additional file 1: Table S1). Moreover, its stability was reduced (0.7 log10) when it underwent a series of three freeze–thaw cycles and was significantly different compared to that in GTS with HG. The only difference between the two formulations in the freeze–thaw cycle test was the presence of HG; therefore, we speculate that the potential cryoprotective features of HG used in this study in the freeze–thaw process positively impacted viral stability. The cryoprotective features of HG have been previously reported with immune biomarkers [26] and food products [27] or probiotics [28, 29], and further study is required to understand these features in the freezing process and their impact on virus stability.

The LRV of AdV-5 was 0.6 log10 following 30 weeks of storage at 4 °C. Previous studies have reported the stability of recombinant adenovirus at 4 °C with an optimal formulation for 12 weeks or 24 months [30, 31]. There are possible factors contributing to the difference in the long-term 24-month (104 weeks) stability in the previous study [31] compared to that at 30 weeks in this study. These factors comprise the use of glass vials for virus storage and surfactants such as polysorbate 80 to additionally prevent virus adsorption to the glass surface or supplementary ingredients in the formulation in the other study, which are most likely required for longer storage times. However, wild-type adenovirus is known to be stable as a nonenveloped virus for multiple years at − 80 °C, but its stability profile at ambient temperatures, such as 4 °C and 25 °C, is poorly known. In fact, the wild-type adenovirus used in this study is reported by the manufacturer to significantly drop in infectious titer following 12 months of storage at − 20 °C. Another study wherein the stability of an adenovirus strain in a lyophilized formulation lasted 24 weeks at 4 °C [32] did not show greater stability than the stability over 30 weeks in liquid formulation in this study. Moreover, the adenovirus remained stable at 25 °C following 8 weeks of storage compared to 1 month (4 weeks) at 30 °C in the previous study. The improvement in virus stability, including for vaccine protection and oncolytic virus therapy, would alleviate the burdens of cold chain and the required cost.

Neither the PIV nor the RV models remained stable for 3 weeks of storage at 25 °C, in contrast to the DNA virus models used in this study. RV outer capsid components play an essential role in virus stability, and the inner core appears to be more thermostable than the outer capsid. The outer capsid protein σ3 preserves infectivity by stabilizing the μ1 protein, which functions to penetrate the host cell membrane [33, 34], and its alteration by chemical or physical agents likely influences virus infectivity [35, 36]. However, a higher infective stock virus titer may have improved the PIV and RV stability results because one of the factors of virus storage stability is the stock virus titer [23, 37]. In fact, PIV and RV stock titers were one thousand times lower than the BHV and AdV virus stock titers, which indicated that they were stable in the HG liquid formulation. Hence, the poor stability profile of PIV and RV in HG is most likely a result of low virus stock titers rather than the developed HG liquid formulation. To confirm this assertion, another new virus stock was prepared to attain a higher titer for RV. The evaluation of stability with a higher titer in the same HG formulation revealed better stability compared to the stability with the lower virus titer (Additional file 1: Table S2).

Interestingly, among the HG samples, those that resulted from basic extraction (alkaline-treated) showed better results than the sample obtained by the acid extraction method (acid-treated) with the BHV-1 model, and an interpretation of this finding would require further investigation. In general, the pI (isoelectric point) values of the alkaline-treated and acid-treated gelatin are low (acidic) and high (basic), respectively. These HG samples with pIs around 5 or 8 were produced by alkaline and acid extraction methods, respectively. The pI of herpesvirus is reported to be approximately 9.6 [38]. There is a possible electrostatic interaction between the HG with a pI of 5 and the herpesvirus to maintain virus surface protein stability during storage at the physiological pH. These results should be interpreted with caution. It is too preliminary to conclude that pIs are a factor affecting virus stability. Further studies are needed to explore the potential effect of the gelatin pI on virus stability.

Nevertheless, this research has some limitations. The stability profiles of other viruses, including PIV, RV, and AdV, with known gelatin parameters, such as MW, concentration, or extraction method, were not evaluated. Additionally, only the stability of BHV was evaluated in gelatin-free buffer or in freeze–thaw stability tests. Moreover, the effect of the current formulation on a vaccine model or genetically modified virus such as the oncolytic virus for potential immunogenicity retention or thermal stability were not evaluated and would be examined in a future study.

## Conclusion

In summary, this study included the examination of the stability of four relevant viruses (enveloped/nonenveloped RNA/DNA viruses) in HG liquid formulation for storage in ambient environments. All virus models remained stable for 8 weeks, and BHV and AdV showed extended stability in the liquid formulation after 30 weeks of storage at 4 °C in 5% HG. The hydrolyzed gelatin used to develop the liquid formulation for all four model viruses was of pharmaceutical grade, had very low levels of endotoxin (< 10 EU/g) and would be relevant for virus and medical research studies and applications.

## Supplementary Information

**Additional file 1: Table S1**. Eight-week storage stability of BHV in GTS buffer at 25 °C, 4 °C and -80 °C. **Table S2**. Stability of RV with two different virus stock titers (LRV ± STD).

## Data Availability

All data and material generated or analyzed in this study are available upon reasonable request and can be provided by Francois Marie Ngako Kadji (fr-ngakokadji@nitta-gelatin.co.jp) or Katsuro Hagiwara (k-hagi@rakuno.ac.jp).

## Abbreviations

HG: Hydrolyzed gelatin
BHV: Bovine herpes virus
PIV: Parainfluenza virus
RV: Reovirus
AdV: Adenovirus
LRV: Log reduction value
MW: Molecular weight
rHSA: Recombinant human serum albumin
HSV: Herpes simplex virus
