# Supplementary material for: Stability of enveloped and nonenveloped viruses in hydrolyzed gelatin liquid formulation
Source: Virol J. 2022 May 27;19:94. doi: 10.1186/s12985-022-01819-w (PMC9137439; doi:10.1186/s12985-022-01819-w)
Supplement: Supplementary file 1 — Additional file 1: Table S1. Eight-week storage stability of BHV in GTS buffer at 25 °C, 4 °C and -80 °C. Table S2. Stability of RV with two different virus stock titers (LRV ± STD). [file 12985_2022_1819_MOESM1_ESM.docx]

| Supplementary Table 1. Eight-week thermal stability of BHV-1 in GTS buffer (LRV ± SD) | | | |
| --- | --- | --- | --- |
| Storage period | 25°C | 4°C | -80°C |
| 3 weeks | 3.4 ± 0.2 | 1.8 ± 0.2 | 0.4 ± 0.2 |
| 8 weeks | 5.9 ± 0.1 | 2.8 ± 0.1 | 0.2 ± 0.2 |

LRV: Log reduction value, SD: Standard deviation

| Supplementary table 2. Stability of RV with two different virus stock titers (LRV ± SD) | | | | |
| --- | --- | --- | --- | --- |
| Virus titers | 2x10^5.2^ TCID50/ml | | 2x10^8.0^ TCID50/ml | |
| Temperatures | 3 weeks | 8 weeks | 3 weeks | 8 weeks |
| -80 ℃ | 1.1 ± 0.0 | 1.0 ± 0.2 | 0 ± 0.2 | 0± 0.21 |
| 4 ℃ | 0.3 ± 0.1 | 0.8 ± 0.1 | 0 ± 0.1 | 0.56± 0.07 |
| 25 ℃ | 1.2 ± 0.1 | 1.6 ± 0.3 | 0 ± 0.2 | 0.6± 0.07 |
